# The Effect of Training on Participant Adherence With a Reporting Time Frame for Momentary Subjective Experiences in Ecological Momentary Assessment: Cognitive Interview Study

**DOI:** 10.2196/28007

**Published:** 2021-05-26

**Authors:** Cheng K Fred Wen, Doerte U Junghaenel, David B Newman, Stefan Schneider, Marilyn Mendez, Sarah E Goldstein, Sarah Velasco, Joshua M Smyth, Arthur A Stone

**Affiliations:** 1 Dornsife Center for Self-Report Science University of Southern California Los Angeles, CA United States; 2 Department of Psychiatry University of California, San Francisco San Francisco, CA United States; 3 Department of Biobehavioral Health Pennsylvania State University University Park, PA United States; 4 Department of Psychology University of Southern California Los Angeles, CA United States

**Keywords:** ecological momentary assessment, EMA, cognitive interview, participant training, reporting period

## Abstract

**Background:**

Ecological momentary assessment (EMA) has the potential to minimize recall bias by having people report on their experiences in the moment (momentary model) or over short periods (coverage model). This potential hinges on the assumption that participants provide their ratings based on the reporting time frame instructions prescribed in the EMA items. However, it is unclear what time frames participants actually use when answering the EMA questions and whether participant training improves participants’ adherence to the reporting instructions.

**Objective:**

This study aims to investigate the reporting time frames participants used when answering EMA questions and whether participant training improves participants’ adherence to the EMA reporting timeframe instructions.

**Methods:**

Telephone-based cognitive interviews were used to investigate the research questions. In a 2×2 factorial design, participants (n=100) were assigned to receive either basic or enhanced EMA training and randomized to rate their experiences using a momentary (at the moment you were called) or a coverage (since the last phone call) model. Participants received five calls over the course of a day to provide ratings; after each rating, participants were immediately interviewed about the time frame they used to answer the EMA questions. A total of 2 raters independently coded the momentary interview responses into time frame categories (Cohen κ=0.64, 95% CI 0.55-0.73).

**Results:**

The results from the momentary conditions showed that most of the calls referred to the period during the call (57/199, 28.6%) or just before the call (98/199, 49.2%) to provide ratings; the remainder were from longer reporting periods. Multinomial logistic regression results indicated a significant training effect (*χ*^2^_1_=16.6; *P*<.001) in which the enhanced training condition yielded more reports within the intended reporting time frames for momentary EMA reports. Cognitive interview data from the coverage model did not lend themselves to reliable coding and were not analyzed.

**Conclusions:**

The results of this study provide the first evidence about adherence to EMA instructions to reporting periods and that enhanced participant training improves adherence to the time frame specified in momentary EMA studies.

## Introduction

### Background

In recent decades, behavioral and psychological researchers have increasingly used ecological momentary assessment (EMA) [1,2] and similar methods (eg, the experience sampling method [3,4]) to measure subjective experiences in daily life. In contrast to traditional self-report instruments, in which participants are asked to provide global evaluations or to retrospectively summarize their experience over a period (eg, 30 days), EMA studies typically ask respondents to answer questions about their experiences just before they were prompted or over short periods. Repeated assessments of participant experiences within a day and over multiple days afford the opportunity to examine various within-person processes [5-7] and changes that occur over short periods [8]. Moreover, by assessing participants in their everyday environments, EMA data are thought to be ecologically valid and to have reduced recall bias [9,10].

When inquiring about participant experiences, there are two commonly implemented approaches: the momentary model and the coverage model [1]. Studies that use the momentary model typically aim to capture participant experiences at the current moment or at the moment just before they were prompted. For example, studies assessing happiness at the moment may use questions such as, “How happy were you feeling right before the prompt?” In contrast, studies implementing a coverage model do not mean to capture current experience but instead capture experiences over a relatively short period. For example, a coverage approach might instruct respondents to report their experiences over the last 30 minutes or since the last time they were prompted (eg, “How happy were you, overall, since the last time you were beeped?”). Both momentary and coverage models have been used in EMA studies to assess a variety of experiences, including affective states [11,12], hunger [13,14], and pain [15,16].

An important assumption of the EMA data is that participants adhere to the stated time frame for their self-report ratings. However, there is no current evidence that participants follow the time frame instructions as intended by the researchers. When asked to report on their experience *right before the prompt*, some participants may provide an overall rating of their experiences over longer periods extending beyond the current moment [17]. Similarly, when asked to report about their experience *since the last prompt*, some participants may only provide information about a portion of the prescribed time frame. A recent study on the time frames that participants used to complete end-of-day diaries illustrates the possibility that people may not follow the designated reporting instructions [18]. Participants were randomized to one of four versions of an end-of-day question meant to assess their affective states over the course of the day. They subsequently indicated the periods they considered while making their ratings. Although all instructions directed participants to rate their affective states over the course of the day (defined in several ways), participants reported using a wide range of time frames, many of which were not consistent with the intended period. For example, only 34.7% (49/141) of individuals reported using time frames consistent with the instructions “In the last day, I felt...” versus 96.5% (141/146) who were consistent when the instructions were “Today, I felt...” Although the instructional wording was similar, the time frames generated by the two instructions varied significantly and suggested that attention should be paid to participants’ interpretations of the diary instructions.

### Objectives

Pertinent to this study, the findings highlight the possibility that participants may report what *makes sense* to them based on the contextual information available in the survey [10] or on conversational norms [19]. This could be problematic as participants’ self-report ratings might not reflect their experiences from a time frame that was intended by the specific research protocol. Interpreting data from outside of the intended reporting time frame could further introduce errors when examining the relationship between EMA data and data from other sources (eg, accelerometers). To our knowledge, there has been no research on how well participants adhere to the time frame instructions used in EMA studies. Therefore, the primary aim of this study is to describe the time frame participants use when answering both the momentary and coverage EMA questions. Owing to the methodological challenges that we experienced when analyzing the reported time frame data for the coverage model in this study (which are described in detail later in this report), we only report results pertinent to the momentary EMA model.

One factor that could influence participants’ adherence to the designated time frame is training. Providing training is a common practice in EMA studies to minimize participant confusion and improve data quality in EMA study protocols. Existing evidence from daily diary studies suggests that training can reduce missing data and improve the internal consistency of diary ratings [20] and that iterative training (eg, providing feedback during the training) is helpful in guiding participants to report on their momentary experience more precisely [21]. A recent systematic review of EMA studies also suggests the importance of providing comprehensive training, including providing examples and opportunities to practice before data collection [22]. However, there is no evidence pertaining to the effectiveness of training on whether participants adhere to the time frame instructions used in the EMA. Therefore, the secondary aim of this study is to evaluate (compared with participants training with *basic instructions* that included just the study procedure) whether training with *enhanced instructions*, which included the opportunity for practice and feedback from the research team in addition to basic instructions, is more effective in generating participant self-reports that are consistent with the intended reporting time frame.

## Methods

### Participant Recruitment

Study participants were recruited through Amazon Mechanical Turk (MTurk) between October 2 and October 18, 2019. The study invitation was only available to registered MTurk workers who had already completed a minimum of 500 approved human intelligence tasks, had a minimum human intelligence task approval rate of 99%, and lived in the Pacific Time (PT) time zones. MTurk workers who met these qualifications were provided with a link to a web-based screener survey in which they provided demographic information and answered five questions that determined their eligibility to participate in the study. Eligibility criteria included being at least 21 years of age, living in the PT time zone, being fluent in English, having access to a phone, and being willing and available to receive and answer five phone calls on the day of the study. Participants were limited to those who resided in the PT time zone and had access to a phone because the interviews were conducted over the phone by members of the research team located in this time zone. Eligible participants were then provided with a detailed study description. Interested participants who agreed to participate were asked to provide their contact information (ie, email address and phone number) to the study team. All MTurk workers who participated in the screening survey, regardless of whether they met the eligibility criteria or provided contact information, were compensated with US $0.50 through MTurk. Interested participants who completed the full study protocol, which included the five phone calls on the subsequent day, were compensated with an additional US $10 through MTurk.

### Study Procedure

Participants were randomized to one of four experimental conditions in a 2×2 factorial design, with a reporting time frame factor (momentary or coverage model) and a training condition factor (basic or enhanced training). The *EMA prompts* in this study were administered using phone calls. We decided to administer the EMA prompts using phone calls because it allowed study staff to conduct semistructured cognitive interviews immediately after participants answered the EMA questions to probe for the time frames that participants used, which would not be feasible to use device-based EMA reports. The EMA items asked participants to report their anxiety, happiness, hunger, and pain. These experience domains were chosen because they are emotional and sensory experiences that are commonly assessed in both coverage and momentary model EMA studies but we acknowledge that this is only a small set of the states and behaviors that could have been chosen.

In all experimental conditions, participants received five phone calls from the research team on one weekday (Monday through Friday) between 9 AM and 5 PM PT. A day before the start of the study, participants were reminded via text messages and through email that a study staff member would contact them shortly after 9 AM PT on the next day. The first call was the introductory call, which took place between 9 AM and 10 AM on the day of the study. During this call, participants were introduced to the study procedures and were informed that all conversations were audiorecorded. If a participant initially did not answer the phone for the introductory phone call, the researcher called two additional times that were at least 15 minutes apart before removing the participant from the study. During the introductory call, participants randomized to the *basic training* condition were read a script that described the overall study procedure with some information about the content of the subsequent calls (Multimedia Appendix 1 provides the full script). Participants randomized to the *enhanced training* condition received the same information as those in the *basic training* condition but were also read a more detailed introductory script. The script for the enhanced training included presentation and practice of the questions that would be asked (ie, how happy, anxious, hungry, or how much pain they felt on a scale from 0 [ie, not at all] to 100 [ie, extremely]) and real-time feedback, to ensure that participants understood the rating scale and time frame as intended by the research team (Multimedia Appendix 2 provides full scripts for participants in the momentary model condition with enhanced training and Multimedia Appendix 3 provides full scripts for participants in the coverage model with enhanced training). After the training, participants were informed that they would subsequently receive four cognitive interview calls at random times throughout the day. The participants were instructed not to call back if they missed a call from the researcher.

After the introductory call, the research team divided the rest of the participant’s day (9 AM to 5 PM) into four 2-hour time windows. The subsequent four cognitive interview calls were scheduled to occur at a randomly selected time within each of these time windows, with a restriction such that no two calls occurred within 45 minutes of each other. If a participant did not answer the phone, a member of the research team called two additional times that were at least 15 minutes apart. All calls were conducted in a semistructured manner. At the beginning of each phone call, the participants were asked for permission to start recording the conversation. After they agreed, participants were asked to provide a rating for one of the four experience domains (ie, level of pain, hunger, anxiousness, and happiness) on a 0-100 scale. In the momentary condition, the wording of the questions as read aloud to the study participants by a research team member was, “On a scale from zero to one hundred, how XX did you feel right before the phone call (momentary model)/since the last call (coverage model), where zero means not at all XX and a hundred means extremely XX?” Participants rated only one of the experience domains (ie, pain, hunger, anxiousness, and happiness) per call, with the order being randomized across calls to minimize the effect of the time of day and question ordering. After the participants provided the rating, they were asked three structured open-ended questions: “What came to mind as you answered this question?” “How did you come up with the rating you provided?” and “There was likely a particular time that you were thinking about when you answered the question. When was that?” After the participants responded to the three open-ended questions, the research staff thanked them and concluded the phone conversation. During the interview, the research staff did not probe participants for further details on their answers, except for clarification purposes, because this interview protocol was intended to solicit participants’ own perspectives and thought processes. All conversations between the research staff and participants were audiorecorded. The introductory call and cognitive interview calls were conducted by different members of the research team to ensure the blinding of participants’ training conditions. All study procedures were approved by the institutional review board of the University of Southern California.

### Coding of Cognitive Interview Data

**Figure 1:**
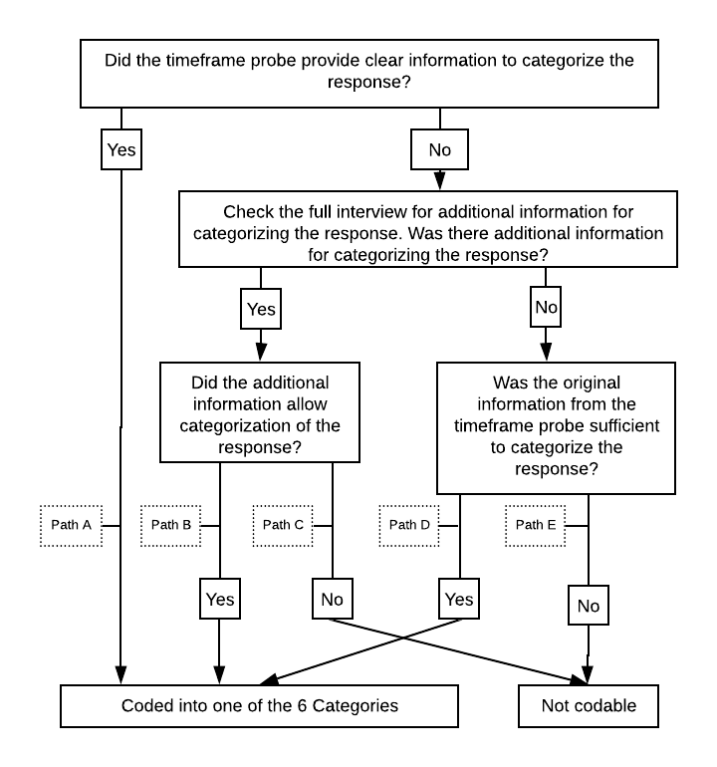
Decision flowchart for categorizing participants’ reporting time frame.

The primary aim of this study was to examine the reporting time frames that study participants used for their ratings. A total of two raters (CKFW and DUJ) first reviewed participants’ responses to the time frame probe (ie, the question of “There was likely a particular time that you were thinking about when you answered the question. When was that?”) because this prompt specifically asked respondents what time frame they had in mind when completing their rating. We first describe the coding of the momentary model, and the flowchart in Figure 1 summarizes the coding decision process. Briefly, the two raters independently coded the time frame probe responses in the momentary model condition into one of seven categories: *during the call*, *right before the call*, *within 5 minutes of the call*, *within 5-15 minutes of the call*, *within 15-60 minutes of the call*, *more than 60 minutes prior to the call*, or *not codable* (path A, Figure 1). The intervals for the first six categories were determined based on an initial review of participant responses and were designed to capture the variety of reporting time frames used by the participants. If participants’ responses to the time frame probe did not provide clear information to categorize the response, the coders reviewed the full interview for additional information that could facilitate the coding of the response. For example, when a participant responded to the time frame probe asking about their hunger level with statements such as “I ate something an hour ago,” the raters reviewed the full interview for additional information to determine whether the participant reported their hunger level 60 minutes ago when they ate something or whether the participant reported their hunger level right before the call and simply mentioned eating something an hour ago. If the full interview contained additional evidence that clarified the participant’s response to the time frame probe (eg, participant mentioned “thinking about his lunch when coming up with how hungry he was right before of the interview” when asked how he came up with the rating), then this additional information was used by the raters to code the reporting time frame (path B).

If raters still could not determine the reporting time frame after reviewing all the available information, the participant’s response was categorized as *not codable* (path C). Continuing with the previous example, if the participant mentioned other time frames (eg, *recently* or *in a couple of hours*) in addition to the *an hour ago* response to the time frame probe, then it was not clear which of these time frames was the one used for the rating, and the reporting time frame was therefore designated as *not codable*. If the full interview did not provide additional information, then the time frame categories were coded based on the participant’s response to the time frame probe, provided that it contained sufficient information for coding. Continuing with “I ate something an hour ago,” if the raters did not find additional information from the full interview, then the reporting time frame for this interview was coded as 60 minutes ago (path D); however, if the participant provided a response such as “I ate something not too long ago,” instead of a concrete time point like “an hour ago,” then this interview was rated as *not codable* (path E). Each rater coded all interviews independently before comparing the results. The two raters discussed discrepancies in their coding and attempted to reach a consensus.

We now turn to coding for the coverage model condition. Unlike the momentary model, the two raters found that the responses to the time frame probe in the coverage model were generally vague and challenging to code. For example, respondents provided answers such as “9 AM” when they were instructed to rate their experience since the last phone call. It was not clear whether the participant meant to report their rating at 9 AM (ie, rating at a single time point) or from 9 AM (ie, an aggregated rating based on the whole time between the reported time point and the current time) based on the available information, that is, it was difficult to determine whether the entire period was used for the response. In fact, when designing the interview protocol, we did not anticipate that the responses would be as vague as they were, and additional probes were not included as part of the protocol. After consulting with the full research team, we determined that the reporting time frame for the coverage model could not be reliably coded. Therefore, we eliminated the coverage model data from further considerations in this study.

### Statistical Analyses

A subset of 10% (20/199) of momentary model interviews was randomly selected as training samples [23] for two practice rounds before the remaining interviews were coded. Interrater reliability was assessed with both the raw percentage of agreement and with Cohen κ using the 180 interviews that were not included in the training subset. All disagreements were resolved by consensus after Cohen κ was calculated. The resulting time frame categories were used for the primary aim of the study, which describes the reporting time frame participants used when answering the EMA questions. As an exploratory aim, we tested whether the intensity of self-report ratings (ie, self-reported levels of pain, hunger, anxiousness, and happiness) differed by time frame category using multilevel regression models. Multilevel regression models also included demographic variables, training conditions, sequential call numbers (ie, call #1, #2, #3, and #4), and the experience domain as covariates to control for potential confounding effects.

The time frame categories were also used as the primary dependent variable in multinomial logistic regression models for examining the second aim of the study: to determine whether the probability of certain time frame categories differed by training condition (ie, basic training or enhanced training). Interviews coded as *not codable* were excluded. The analytic model also included demographic variables (ie, age, gender, race, education level, marital status, and annual household income level) as well as sequential call numbers (ie, call #1, #2, #3, and #4) and the experience domain (ie, pain, hunger, anxiousness, and happiness) as covariates to control for potential confounding effects. Interactions among the covariates were not explored, as this went beyond the main goals of the study. Models with categorical outcomes (time frame categories) were conducted using Proc GENMOD, and models with continuous outcomes (self-report ratings) were conducted using Proc MIXED in SAS (version 9.4; SAS Institute).

## Results

### Participant Characteristics

Of the 672 MTurk workers who accessed the screening survey, 94 exited the survey before the study description was presented, 388 did not meet the eligibility criteria (one was aged <21 years, 67 did not reside in the PT time zone, 125 reported not having access to a phone, and 195 did not wish to receive five phone calls), and 47 were not interested in participating. All 143 eligible and interested participants provided their contact information to the research team, but 43 did not respond to contact attempts made by the research team for the introduction call. This group of people was, on average, younger (mean age 35.1, SD 8.9 years) than the 100 who responded to contact attempts (mean age 40.1, SD 12.8 years; t_141_=−2.34, two-tailed; *P*=.02) but did not differ in other demographic characteristics. The participant characteristics of the remaining 100 participants who participated in the full study protocol are presented in Table 1. The participant characteristics did not significantly differ between the four experimental conditions. Participants who were randomized to the basic training conditions spent an average of 145.9 (SD 27.4) seconds, whereas those randomized to the enhanced training conditions spent an average of 430.6 (SD 62.2) seconds on the introductory call. Of 400 scheduled calls for cognitive interviews, 359 (89.7%) were answered at the first contact, 29 (7.2%) were answered at the second attempt, and 11 (2.8%) were answered at the third and final attempt. The research team was not able to reach one participant for 1 (0.3%) occasion after three attempts of contact.

**Table 1:** Participant demographic characteristics.

Variable		Population (N=100)	Momentary model			Coverage model	
			Basic training (n=25)	Enhanced training (n=25)	Basic training (n=25)		Enhanced training (n=25)
Age (years), mean (SD)		40.1 (12.8)	36.4 (9.2)	42.6 (13.3)	38.0 (11.9)		43.3 (15.4)
**Gender, n (%)**							
	Female	50 (50)	14 (56)	12 (48)	14 (56)		10 (40)
	Male	50 (50)	11 (44)	13 (52)	11 (44)		15 (60)
**Ethnicity, n (%)**							
	Non-Hispanic	83 (83)	19 (76)	21 (84)	21 (84)		22 (88)
	Hispanic	17 (17)	6 (24)	4 (16)	4 (16)		3 (12)
**Race, n (%)**							
	African American	2 (2)	0 (0)	1 (4)	1 (4)		0 (0)
	Asian	9 (9)	1 (4)	2 (8)	2 (8)		4 (16)
	Native American	2 (2)	0 (0)	1 (4)	0 (0)		1 (4)
	Mixed	12 (12)	3 (12)	1 (4)	4 (16)		4 (16)
	White	75 (75)	21 (84)	20 (80)	18 (72)		16 (64)
**Marital status, n (%)**							
	Never married	38 (38)	10 (40)	7 (28)	9 (36)		12 (48)
	Living with partner	11 (11)	4 (16)	4 (16)	1 (4)		2 (8)
	Married	34 (34)	7 (28)	9 (36)	12 (48)		6 (24)
	Divorced	12 (12)	3 (12)	3 (12)	3 (12)		3 (12)
	Separated	1 (1)	0 (0)	1(4)	0 (0)		0 (0)
	Widowed	4 (4)	1 (4)	1(4)	0 (0)		2 (8)
**Education, n (%)**							
	High school graduate	5 (5)	1 (4)	0 (0)	3 (12)		1 (4)
	Some college	26 (26)	9 (36)	8 (32)	6 (24)		3 (12)
	College graduate	60 (60)	10 (40)	16 (64)	14 (56)		20 (80)
	Master’s degree	6 (6)	5 (20)	1 (4)	0 (0)		0 (0)
	Doctoral degree	3 (3)	0 (0)	0 (0)	2 (8)		1 (4)
**Employment status, n (%)**							
	Employed full time	56 (56)	16 (64)	13(52)	15 (60)		12 (48)
	Employed part time	20 (20)	4 (16)	7 (28)	7 (28)		2 (8)
	Self-employed	7 (7)	2 (8)	3 (12)	0 (0)		2 (8)
	Homemaker	5 (5)	1 (4)	0 (0)	1 (4)		3 (12)
	Retired	6 (6)	0 (0)	2 (8)	1 (4)		3 (12)
	Unemployed	4 (4)	1 (4)	0 (0)	1 (4)		2 (8)
	Unable to work	2 (2)	1 (4)	0 (0)	0 (0)		1 (4)
**Income (US $), n (%)**							
	<20,000	13(13)	8 (32)	2 (8)	2 (8)		1 (4)
	20,000-34,999	16 (16)	3 (12)	5 (20)	3 (12)		5 (20)
	35,000-49,999	20 (20)	4 (16)	5 (20)	7 (28)		4 (16)
	50,000-74,999	18 (18)	4 (16)	2 (8)	4 (12)		8 (32)
	>75,000	33 (33)	6 (24)	11 (44)	9 (36)		7 (28)

### Reliability of Coding Participants Reporting Time Frame

Of the 200 interviews conducted for the momentary condition, one call was excluded because of administrative errors (the call was accidentally not recorded). The two raters independently coded all the remaining 199 interviews. They agreed on 74.9% (134/179) of the interviews, resulting in a *moderate* interrater reliability [24] (Cohen κ=0.64, 95% CI 0.55-0.73). All disagreements were resolved by consensus after Cohen κ was calculated.

### Reporting Time Frame Categories in Momentary Model Conditions

Of the 199 cognitive interviews collected in the momentary model conditions, 57 (28.6%) were coded as *during the call*, 98 (49.3%) were coded as *right before the call*, 10 (5.1%) were coded as *within 5 minutes of the call*, 8 (4.0%) were coded as *within 5-15 minutes of the call*, 7 (3.5%) were coded as *within 15-60 minutes of the call*, 13 (6.5%) were coded as *more than 60 minutes prior to the call*, and 6 (3.0%) were coded as *not codable* (Table 2). The raters reviewed the full interview for additional information in 36.2% (72/199) of calls. After the full interview review, 8.3% (6/72) of interviews remained uncodable. Of those coded as *not codable*, the raters were unable to identify a reporting time frame for four interviews because the participant did not provide sufficient information over the entire interview. The raters were unable to identify a reporting time frame for the remaining two interviews because participants reported multiple and conflicting time frames.

**Table 2:** Reporting time frame categories by training condition (momentary condition).

Reporting time frame categories	Overall (N=199), n (%)	Training condition, n (%)	
		Basic training (n=100)	Enhanced training (n=99)
During call or right now	57 (28.6)	40 (40.0)	17 (17.2)
Just before the call	98 (49.2)	23 (23.0)	75 (75.8)
≤5 min before call	10 (5.0)	5 (5.0)	5 (5.1)
>5 min, but ≤15 min before call	8 (4.0)	7 (7.0)	1 (1.0)
>15 min, but ≤60 min before call	7 (3.5)	7 (7.0)	0 (0.0)
>60 min before call	13 (6.5)	12 (12.0)	1 (1.0)
Not codable	6 (3.0)	6 (6.0)	0 (0.0)

### Distribution of Momentary Reporting Time Frame Category by Training Condition

Multinomial logistic regression models showed a significant effect of training on the momentary reporting time frame categories (*χ*^2^_1_=16.6; *P*<.001). None of the covariates included in the analytic model were significant. The proportions of reporting time frame categories by training assignments are presented in Table 2.

## Discussion

### Principal Findings

Data collected using EMA methods have the potential to provide a fine-grained understanding of a wide range of psychological, behavioral, and medical phenomena as they occur in daily life. Despite the growing interest in EMA for assessing participant experiences, little attention has been paid to whether participants adhere to the reporting time frame instructions in EMA studies. In this study, we examined this question for commonly used EMA items with momentary reporting instructions. Unfortunately, the data were not suitable for examining the question for coverage model EMA items, which is discussed further in the following section. For the momentary condition, the results showed that although nearly half of the EMA data were reported to be from the intended time frame (ie, just before the call), participants also drew on other reporting time frames, including during the call or other time frames before the moment before the call. The study also revealed that compared with a basic training procedure, an enhanced training protocol with detailed explanations and opportunities for practice was effective in improving participants’ adherence to momentary time frame instructions.

First, we consider our inability to reliably code coverage model interviews. Compared with the momentary model, there were many more ways in which a participant could engage in responding to the coverage model questions. When rating their experience under the coverage model, participants could have considered the proximity and duration of a relevant event related to the inquired experiences and could have reported their rating based on a single moment, shorter or longer periods that covered only parts or all the time between two phone calls. Our interview protocol was not fully prepared to handle these complexities, as we used an open-ended approach to elicit responses, and participants’ statements were often too ambiguous to confidently categorize their responses. Future investigations of how participants answer coverage model questions will need to incorporate additional probes in the interview protocol that are designed to solicit more specific information about the proximity and duration of experiences.

In terms of the results for the momentary model, this study provided detailed information about the time frames used in the basic and enhanced training conditions so that readers may consider what *they* view as acceptable adherence to momentary EMA instructions. We grouped together the categories of *during the call/right now*, *just before the call*, and *within 5 minutes of the call* in our discussion in the following paragraph because, in our opinion, these seem to be reasonable time frames for momentary EMA questions. However, other researchers may find this categorization too liberal for their purposes (eg, associating affective states with ambulatory monitoring of heart rate the minute before the prompt) and instead adopt a more stringent rule where only those time frames that included how participants felt *just before the call* are considered valid. Researchers with other goals for their EMA studies may choose to adopt more liberal rules.

For participant training, the results for the momentary data showed that when participants were provided with basic training, only 68.0% (68/100) of the interviews were coded into the three categories of less than 5 minutes before the call, just before the call, and during the call; only 23.0% (23/100) of the interviews were coded as just before the call. In our view, these percentages are less than ideal and suggest that simply stating the intended reporting time frame as part of the EMA questions without more detailed participant training is not sufficient to achieve high adherence rates to the intended time frame. A possible explanation for this could be that instead of considering the literal meaning of *right before the call*, participants may have responded based on conversational norms [10,19]. For example, when a participant was asked to rate their hunger level *right before the call*, perhaps they may have thought that we meant to capture their hunger level around the time they last ate something before the phone call because this information may be more in line with what is worth communicating based on conversational norms in daily life (or what the participant thought the researchers really wanted to know).

When participants were provided with enhanced training before data collection in the momentary condition, significantly more calls (75/99, 76%) had a reporting time frame coded as just before the call and 98% (97/99) had a reporting time frame coded as less than 5 minutes before the call, just before the call, and during the call. This is an excellent level of adherence to the instructions, assuming that a reporting time frame from within 5 minutes of the prompt is appropriate for one’s research goals and argues that researchers should ensure that participants are thoroughly trained in momentary data collection. This is especially important as self-reports for momentary EMA because longer-than-momentary reporting time frames (≥2 hours) have previously been shown to yield systematically higher rating levels compared with immediate ratings [25]. These level differences could bias the EMA reports if a sizable portion of a sample did not adhere to the intended reporting time frames.

Although this study provides new information regarding participants’ self-reported time frames in EMA studies, it has limitations. First, although the cognitive interview phone calls were conducted according to a typical EMA schedule, we acknowledge the possibility of the mode of administration effects. For example, participants’ responses to a telephone interviewer could differ from responses provided without an interviewer. It is possible that participants may tend to provide more socially desirable responses to the interviewer [26]. Another example of the administration effect could be that the study participant picks up subtle and unintended verbal cues from the research team members as they read the items aloud (eg, emphasis on specific parts of the sentence). Both examples may have introduced some bias in the resulting data. Second, it is challenging to verify how and whether the participants made their ratings based on the intended time frame. For example, it is possible that some participants responded with *right before the prompt* as their answer to the time frame probe by simply repeating the time frame instruction from the question regardless of the time frame they actually used. Future studies extending this line of investigation could incorporate additional interview procedures (eg, using the *think-aloud* method) that solicit the details of the thought processes leading up to their ratings for both the momentary and coverage models. A third limitation is that we only assessed the time frame associated with each domain once per participant. An essential characteristic of EMA is that it is comprised repeated assessments of the same constructs, and this feature of EMA studies may alter the time frames used as a study progresses. For this reason, the results presented here may only be generalizable to the first few EMA prompts. Future studies that inquire about participants’ reporting time frames on many occasions would provide additional information as to whether participants improve their adherence to the desired time frame or drift into broader time frames throughout study participation. Conducting cognitive interviews in a random and intermittent manner within an existing EMA protocol may potentially be an opportunity to expand on this question. The fourth limitation is the generalizability of the results to participants recruited from the general public. The training effect may be more robust in MTurk workers than in the general population, possibly because MTurk workers tend to be better educated [27] and more attentive to instructions [28]. Future studies that involve participants from a more diverse pool of participants would be able to further expand on the training effect documented in this study.

### Conclusions

In summary, this study provides evidence that participants do not reliably use the momentary time frame intended for EMA protocols when brief instructions are provided; rather, they provide evidence that respondents often appear to use longer periods. The results also indicate that training participants with detailed time frame definitions and providing opportunities to practice EMA reports during training substantially improved participants’ adherence to the time frame instructions. Adherence levels in coverage EMA were not codable in this study; therefore, this remains to be a question for future research.

## Appendix

Multimedia Appendix 1 Actual training script for groups assigned to receive basic training for both momentary and coverage model conditions.

Multimedia Appendix 2 Actual training script for groups assigned to enhanced training in the momentary model.

Multimedia Appendix 3 Actual training script for groups assigned to enhanced training in the coverage model.

## Abbreviations

EMA: ecological momentary assessment
MTurk: Mechanical Turk
PT: Pacific Time
