# Supplementary material for: The Effect of Training on Participant Adherence With a Reporting Time Frame for Momentary Subjective Experiences in Ecological Momentary Assessment: Cognitive Interview Study
Source: JMIR Form Res. 2021 May 26;5(5):e28007. doi: 10.2196/28007 (PMC8190649; doi:10.2196/28007)
Supplement: Multimedia Appendix 1 [file formative_v5i5e28007_app1.docx]

Appendix 1: Actual Training Script for Groups Assigned to Receive Basic Training for Both Momentary and Coverage Model Conditions

“Hello, this is *name of research assistant* from the Center for Self-Report Science. Thank you very much for agreeing to participate in our study. This phone call and the remaining 4 phone calls throughout the day will be audio-recorded. Do I have your permission to begin recording? During this phone call, I would like to describe to you what is involved in participation and answer any questions that you may have about the study. Is now a good time to talk?

[If yes] Great, let’s begin.

[If no, Ok, I’ll call you back in a bit]

As you saw from the study description, you will participate in this study for one day, which is today, and receive 5 phone calls throughout the day. The first phone call is to introduce you to the study procedures and this is the phone call that we are doing now. The remaining 4 phone calls will be interspersed throughout the day between now and 5 p.m. this evening. During each of these phone calls, we will ask you about your experiences, such as your mood and physical sensations. The phone calls are relatively brief and will only take about 5 minutes each. It is important for you to know that these phone calls will happen randomly throughout the day, which means we will not schedule specific times with you and you will need to be near your phone and available to speak with us between now and 5 p.m. this evening. We understand that it is possible that you might need to miss a phone call, for example, if you are driving. There is no need to call us back. If we cannot reach you, we will try calling you again at another time. However, please know that it is very important that we complete all four phone calls today between now and 5 p.m. this evening. Do you have any questions about the study? Are there particular times during the day where we should not call you? Is this number the best number to reach you today?

[If not, get the best phone number from participant.]

[If yes] Great, other members of the research team will be calling you for the four phone calls. Thank you for speaking with us.”
