# Supplementary material for: The Effect of Training on Participant Adherence With a Reporting Time Frame for Momentary Subjective Experiences in Ecological Momentary Assessment: Cognitive Interview Study
Source: JMIR Form Res. 2021 May 26;5(5):e28007. doi: 10.2196/28007 (PMC8190649; doi:10.2196/28007)
Supplement: Multimedia Appendix 2 [file formative_v5i5e28007_app2.docx]

Appendix 2: Actual Training Script for Groups Assigned to Enhanced Training in the Momentary Model

“Hello, this is *name of research assistant* from the Center for Self-Report Science. Thank you very much for agreeing to participate in our study. This phone call and the remaining 4 phone calls throughout the day will be audio-recorded. Do I have your permission to begin recording? During this phone call, I would like to describe to you what is involved in participation and answer any questions that you may have about the study. Is now a good time to talk?

[If yes] Great, let’s begin.

[If no, Ok, I’ll call you back in a bit]

As you saw from the study description, you will participate in this study for one day, which is today, and receive 5 phone calls throughout the day. The first phone call is to introduce you to the study procedures and this is the phone call that we are doing now. The remaining 4 phone calls will be interspersed throughout the day between now and 5 p.m. this evening. During each of these phone calls, we will ask you about your experiences, such as your mood and physical sensations. The phone calls are relatively brief and will only take about 5 minutes each. It is important for you to know that these phone calls will happen randomly throughout the day, which means we will not schedule specific times with you and you will need to be near your phone and available to speak with us between now and 5 p.m. this evening. We understand that it is possible that you might need to miss a phone call, for example, if you are driving. There is no need to call us back. If we cannot reach you, we will try calling you again at another time. However, please know that it is very important that we complete all four phone calls today between now and 5 p.m. this evening. Do you have any questions about the study? Are there particular times during the day where we should not call you? I would now like to give you more detail about the types of questions that we will be asking you about and what information we would like to get from you during this study.

In this study, we are interested in learning more about people’s experiences *in the moment*. This means that we are not interested in learning about how you generally feel. Instead, we would like to know about your experiences *right before* each of the four phone calls. To be specific, during today’s phone calls we will ask you how happy you felt, how anxious you felt, how much pain you felt, and how hungry you were. You get one topic during each phone call. When you answer the questions, we would like for you to think about how you felt immediately before we started the phone call. You might feel inclined to think about a longer time period, for example, how happy or anxious you felt five minutes before the phone call or all day up until this phone call or during a specific event that occurred during the day. This is not what we are looking for. We only want you to focus on how you felt right the second before the phone call. Do you have any questions?

We also want you to know that we will ask you to rate your experiences in that moment on a 0 to 100 rating scale during the phone call. 0 would mean that you were not at all happy, anxious, hungry, or in pain right before we called you and 100 would mean that you were extremely happy, anxious, hungry, or felt extreme pain right before we called you. You can choose any number between 0 and 100 to best describe your experience.

Let’s go through each of these to make sure you know how to use the rating scale.

Imagine you hit your toe five minutes before the phone call, but by the time the phone rang, you hardly felt it any longer. How would you rate your pain right before we called you on a scale from 0 to 100, where 0 would mean that you were not at all in pain and 100 would mean that you were in extreme pain? Did you only consider how you felt immediately before the phone call? [If yes: Great! We only want you to consider the moment before the phone call, not the entire five minutes or a longer time period. If no: Try to only think of how you felt the moment right before the phone call. Do not try to think back several minutes or even longer.

Now, imagine that right before the phone rang, you won an expenses paid vacation. How would you rate your happiness on this scale from 0 to 100 for that moment? Again, 0 would mean that you were not at all happy and 100 would mean that you were extremely happy. Did you only consider how you felt immediately before the phone call?

Now, imagine that you had just finished a full meal when the phone rang. How hungry would you say you were right before we called you on a scale from 0 to 100? Again, 0 would mean that you were not at all hungry and 100 would mean that you were extremely hungry. Did you only consider how you felt immediately before the phone call?

Finally, imagine that you were on a pleasant and relaxing hike and just saw a rattle snake next to your foot when the phone rang. How anxious would you say you were right before we called you on a scale from 0 to 100? Again, 0 would mean that you were not at all anxious and 100 would mean that you were extremely anxious. Did you only consider how you felt immediately before the phone call?

Do you have any questions about using this rating scale?

Finally, we will also be asking you how you came up with your rating, so be prepared to tell us what you were thinking about when you chose a particular number on the scale. Do you have any questions? Great, this is all that is involved in your participation and we are ready to start the study. Is this number the best number to reach you today?

[If not, get the best phone number from participant.]

[If yes] Great, other members of the research team will be calling you for the four phone calls. Again, please remember that we are only interested in your experiences *the moment before* each phone call. Thank you for speaking with us.”
