# Supplementary material for: The Effect of Training on Participant Adherence With a Reporting Time Frame for Momentary Subjective Experiences in Ecological Momentary Assessment: Cognitive Interview Study
Source: JMIR Form Res. 2021 May 26;5(5):e28007. doi: 10.2196/28007 (PMC8190649; doi:10.2196/28007)
Supplement: Multimedia Appendix 3 [file formative_v5i5e28007_app3.docx]

Appendix 3: Actual Training Script for Groups Assigned to Enhanced Training in the Coverage Model

“Hello, this is [insert name] from the Center for Self-Report Science. Thank you very much for agreeing to participate in our study. This phone call and the remaining 4 phone calls throughout the day will be audio-recorded. Do I have your permission to begin recording? [If yes, start recording] During this phone call, I would like to describe to you what is involved in participation and answer any questions that you may have about the study. Is now a good time to talk? [If yes] Great, let’s begin. [If no, Ok, I’ll call you back in a bit] As you saw from the study description, you will participate in this study for one day, which is today, and receive 5 phone calls throughout the day. The first phone call is to introduce you to the study procedures and this is the phone call that we are doing now. The remaining 4 phone calls will be interspersed throughout the day between now and 5 p.m. this evening. During each of these phone calls, we will ask you about your experiences, such as your mood and physical sensations. The phone calls are relatively brief and will only take about 5 minutes each. It is important for you to know that these phone calls will happen randomly throughout the day, which means we will not schedule specific times with you and you will need to be near your phone and available to speak with us between now and 5 p.m. this evening. We understand that it is possible that you might need to miss a phone call, for example, if you are driving. There is no need to call us back. If we cannot reach you, we will try calling you again at another time. However, please know that it is very important that we complete all four phone calls today between now and 5 p.m. this evening. Do you have any questions about the study? [If no] Are there particular times during the day where we should not call you? [If no], Great, I would now like to give you more detail about the types of questions that we will be asking you about and what information we would like to get from you during this study.

In this study, we are interested in learning more about people’s experiences from one phone call to the next. This means that we are not interested in learning about how you generally feel. Instead,

we would like to know about your experiences since the last call you received from us. To be specific, during today’s phone calls we will ask you how happy you felt, how anxious you felt, how much pain you felt, and how hungry you were. You get one topic during each phone call. When you answer the questions, we would like for you to think about how you felt since the last phone call. For example, in the next call we have with you we will ask how you felt since this phone call. You might feel inclined to think about a longer time period, for example, how happy or anxious you felt all day up until this phone call or how you felt during the phone call. This is not what we are looking for in this study. We only want you to focus on the period between each of the four phone calls. Do you have any questions?

[If not] Great, we also want you to know that we will ask you to rate your experiences in between each phone call on a 0 to 100 rating scale. 0 would mean that you were not at all happy, anxious, hungry, or in pain since the last phone call and 100 would mean that you were extremely happy, anxious, hungry, or felt extreme pain since the last phone call. You can choose any number between 0 and 100 to best describe your experience.

Let’s go through each of these to make sure you know how to use the rating scale.

Imagine you hit your toe since the last phone call. At first, your toe hurt badly but by the time the phone rang, you hardly felt it any longer. How would you rate your pain since the last phone call on a scale from 0 to 100, where 0 would mean that you were not at all in pain and 100 would mean that you were in extreme pain? Did you only consider how you felt immediately before the phone call or did you take the whole time period since the last phone call into account? [If the whole time period: Great! We want you to consider the whole time period since the last phone call, not just how you felt right before we called you. If no: Try to think of how you felt since the last phone call taking all of your experiences into consideration.]

Now, imagine that you watched a sad movie since the last phone call but right before the phone rang, you won in the lottery. How would you rate your happiness on this scale from 0 to 100 since the last phone call? Again, 0 would mean that you were not at all happy and 100 would mean that you were extremely happy. Did you consider the whole time period since the last phone call?

Now, imagine that you were a bit hungry since the last phone call but had just finished a full meal when the phone rang. How hungry would you say you were since the last phone call on a scale from 0 to 100? Again, 0 would mean that you were not at all hungry and 100 would mean that you were extremely hungry. Did you consider the whole time period since the last phone call?

Finally, imagine that you were on a hike since the last phone call and just saw a rattle snake next to your foot when the phone rang. How anxious would you say you were since the last phone call on a scale from 0 to 100? Again, 0 would mean that you were not at all anxious and 100 would mean that you were extremely anxious. Did you consider the whole time period since the last phone call? Do you have any questions about using this rating scale? [If no] Great.

Finally, we will also be asking you how you came up with your rating, so be prepared to tell us what you were thinking about when you chose a particular number on the scale. Do you have any questions? [If no] Great, this is all that is involved in your participation and we are ready to start the study. Is this number the best number to reach you today? [If not, get the best phone number from participant.] [If yes] Great, other members of the research team will be calling you for the four phone calls. Again, please remember that we are interested in your experiences since the last phone call. Thank you for speaking with us.”
